# From Cardiac Arrest to Survival: Managing Acute Type A Aortic Dissection With Emergent Surgery

**DOI:** 10.7759/cureus.78231

**Published:** 2025-01-30

**Authors:** Vasileios Leivaditis, Ece Özsoy, Manfred Dahm, Athanasios Papatriantafyllou, Tamas Büki, Nikolaos G Baikoussis

**Affiliations:** 1 Department of Cardiothoracic and Vascular Surgery, Westpfalz-Klinikum, Kaiserslautern, DEU; 2 Department of Cardiac Surgery, Ippokrateio General Hospital of Athens, Athens, GRC

**Keywords:** acute type a aortic dissection, aortic valve replacement, cardiac arrest, emergent surgery, extracorporeal circulation, hypothermic circulatory arrest, multidisciplinary management

## Abstract

Acute type A aortic dissection (AAD) is a life-threatening cardiovascular emergency with extremely high mortality, especially if complicated by cardiac arrest. Early diagnosis and prompt surgical intervention are essential for survival but pose major difficulties in unstable patients. We describe the clinical course of a 68-year-old man with out-of-hospital cardiac arrest due to AAD. Cardiopulmonary resuscitation was performed on-site and was in progress during transport. After achieving return of spontaneous circulation in the emergency department, emergency coronary angiography ruled out coronary artery disease and revealed aneurysmal dilation of the ascending aorta, severe aortic valve regurgitation, and an intimal flap consistent with dissection. The diagnosis of AAD from the aortic root to the iliac arteries, with pericardial and left pleural effusions, was confirmed by total-body computed tomography. Emergent surgical management included the replacement of the dissected ascending aorta with a 28 mm synthetic graft and replacement of the severely regurgitant aortic valve with a 21 mm bioprosthesis. The procedure was carried out with full circulatory arrest and axillary cannulation. The patient’s postoperative course was complicated by coagulopathy and slow gradual neurological improvement, but ultimately, he had no evidence of ischemic or hemorrhagic brain injury. He was discharged in stable condition on the 15th postoperative day. Follow-up imaging showed stable chronic dissection of the descending aorta, as well as complete resolution of pleural and pericardial effusions. This case highlights the complexities of diagnosing and managing AAD in a patient presenting with cardiac arrest. It also demonstrates the importance of multidisciplinary collaboration, timely imaging, and advanced surgical techniques in overcoming the significant challenges associated with this critical condition.

## Introduction

Acute type A aortic dissection (AAD) is a life-threatening condition that necessitates immediate diagnosis and surgical treatment. It occurs due to an intimal tear in the ascending aorta that forms a false lumen potentially resulting in life-threatening complications including cardiac tamponade, acute aortic valve regurgitation, or malperfusion syndrome [1]. It is a rare but severe disorder of the aorta and appears in around 3.5 out of 100,000 individuals each year [2]. AAD has a high mortality rate and, in the acute phase, nearly one to two percent of patients die per hour, if not managed in time during the acute phase of its manifestation [3].

The clinical presentation of AAD can widely differ, ranging from severe chest pain [1] to hemodynamic instability or cardiac arrest [4,5]. Hemodynamic instability and the need for cardiopulmonary resuscitation (CPR) severely reduce the prognosis of these cases [4,5]. When patients present in extremis, diagnosis may be particularly difficult, as advanced imaging is often delayed due to the need for immediate resuscitation of cardiac arrest. Computed tomography (CT) is crucial for confirming the diagnosis and for ascertaining the extent of dissection, but intervention decisions must often be made urgently in unstable patients [6].

Surgical management usually includes the replacement of the torn ascending aorta with a prosthetic graft along with the management of related problems such as aortic valve insufficiency. In the face of the failure of contemporary surgical techniques and perioperative care, to render the procedures safe, its high-risk nature has not altered in instances of peri-arrest physiology, where additional challenges including coagulopathy, organ dysfunction, and potential neurological injury may further complicate the complication [1,6].

This report describes the case of a 68-year-old male patient with AAD who developed out-of-hospital cardiac arrest. We here attempt to analyze the challenges, which arise regarding diagnosis and emergency surgical management in the context of rapid decision-making and multi-disciplinary care, in order to achieve a successful outcome.

## Case presentation

A 68-year-old man with no known cardiovascular history collapsed suddenly while walking outside with his wife on a cold afternoon. Emergency medical services (EMS) were immediately called and arrived about 20 minutes later, discovering the patient was in cardiac arrest. CPR was begun at the scene and was further continued during transportation to the hospital. On arrival at the emergency department (ED), approximately 40 min after the initial collapse, the patient was still in cardiogenic shock requiring continued resuscitation efforts. After extended CPR, return of spontaneous circulation (ROSC) occurred.

Initial evaluation

**Figure 1:**
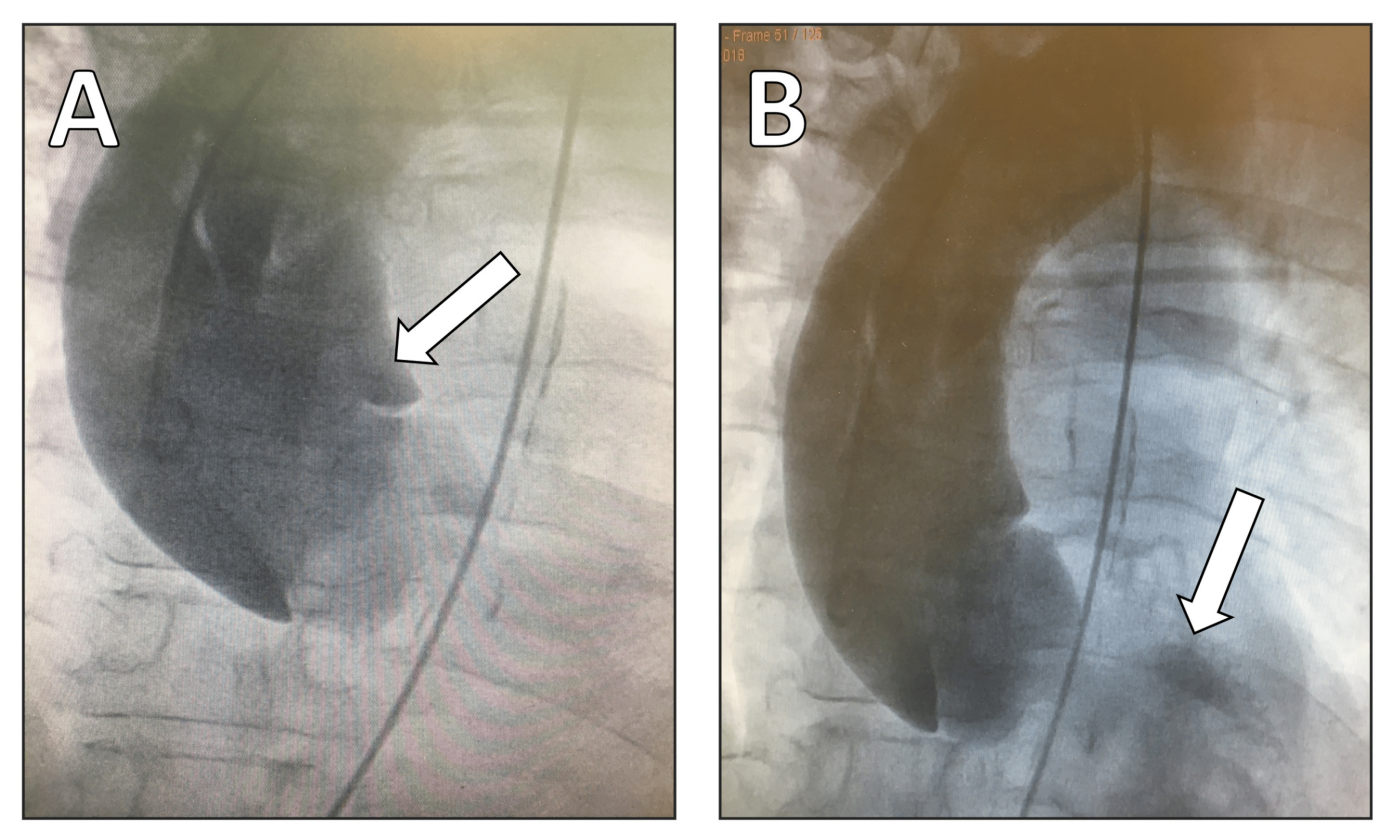
(A) Aneurysmal dilatation of the ascending aorta with an intimal flap and a possible entry point located above the ostium of the left main coronary artery (LM). (B) Contrast medium visible in the left ventricle due to significant aortic valve regurgitation.

In the ED, the patient underwent an initial evaluation that included an electrocardiogram (ECG), which demonstrated only nonspecific changes of concern for either acute coronary syndrome (ACS) or another potentially serious cardiovascular event. Due to initial suspicion of an obstructive left main coronary artery lesion, he underwent urgent, non-elective coronary angiography and aortography. Normal coronary ostia on angiography ruled out coronary artery occlusion as a cause of arrest. However, there was marked aneurysmal dilation of the ascending aorta and an intimal flap consistent with AAD (Figure 1A). Moreover, a severe asymmetric aortic valve regurgitation was present as seen by the retrograde flow of contrast medium into the left ventricle (Figure 1B).

Imaging findings

**Figure 2:**
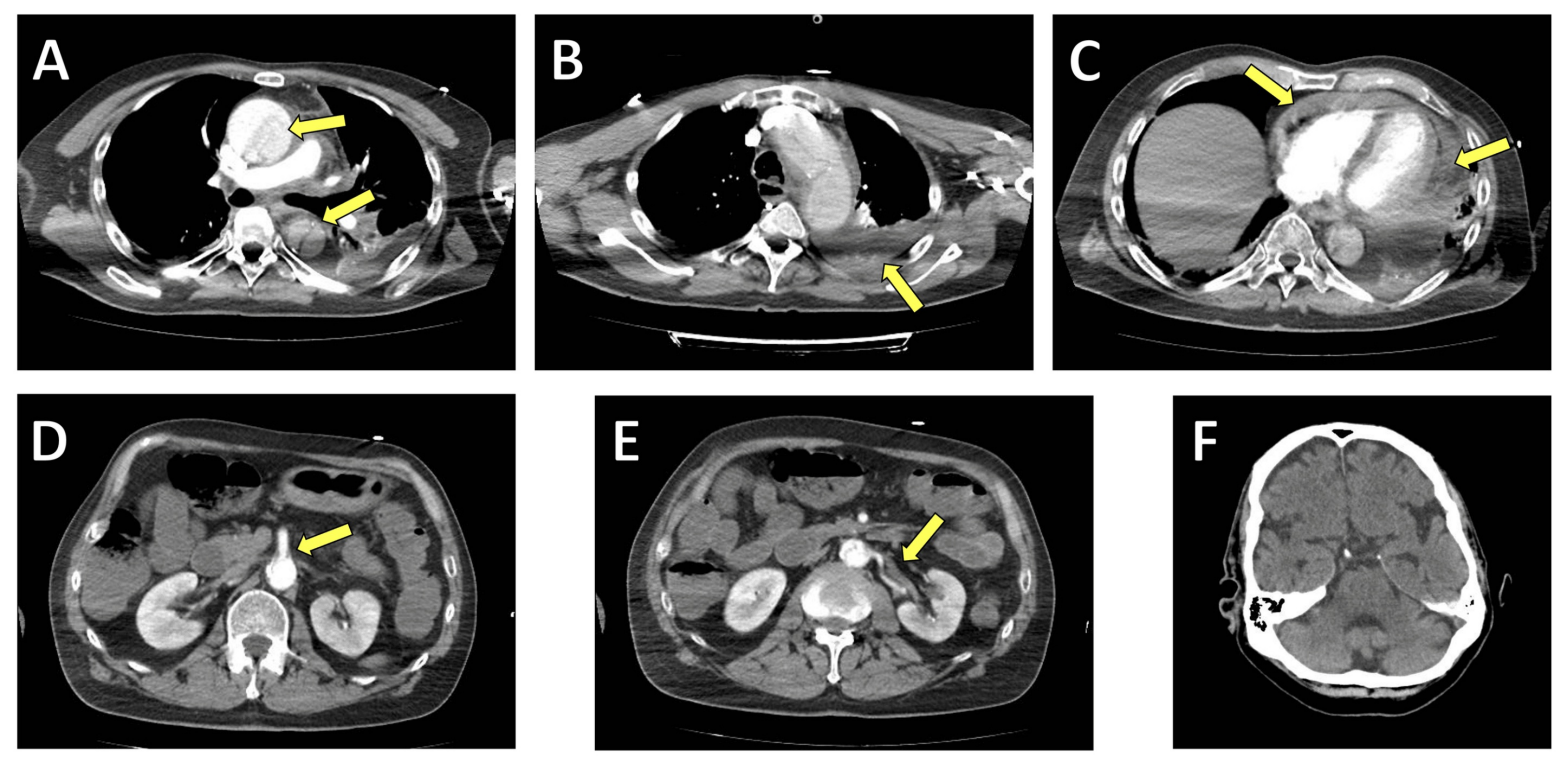
Preoperative imaging findings: A) Acute type A aortic dissection with a dissected ascending aorta (arrow). (B) Left pleural effusion (arrow). (C) Pericardial effusion (arrow) caused by endopericardial rupture of the aorta. (D) Abdominal CT scan showing dissection extending into the abdominal aorta, with involvement of the superior mesenteric artery (arrow). (E) Dissection of the renal arteries. (F) Preoperative brain CT scan performed as a baseline due to cardiac arrest and the uncertain prognosis for postoperative neurological recovery.

A total-body computed tomography (CT) scan was carried out for better assessment of the extent of the pathology and to rule out relevant complications. Chest CT demonstrated the AAD extending from the aortic root across the ascending aorta, with a pericardial effusion likely resulting from an endopericardial rupture (Figure 2). Left pleural effusion was also noted. Abdominal CT revealed extension of the dissection into the abdominal aorta with involvement of the superior mesenteric and renal arteries. Brain CT was also performed preoperatively due to the prolonged cardiac arrest and unclear neurological prognosis, showing no ischemic or hemorrhagic lesions but being interpreted as baseline imaging for postoperative assessments.

Decision for surgery and surgical procedure

In light of the discovery of the extended aortic dissection with severe aortic regurgitation and pericardial effusion in addition to the patient’s clinical instability, an urgent operation was decided. Axillary cannulation for extracorporeal circulation was preferable to maximize access for surgical repair. The patient was subjected to median sternotomy under general anesthesia after fast preparation.

**Figure 3:**
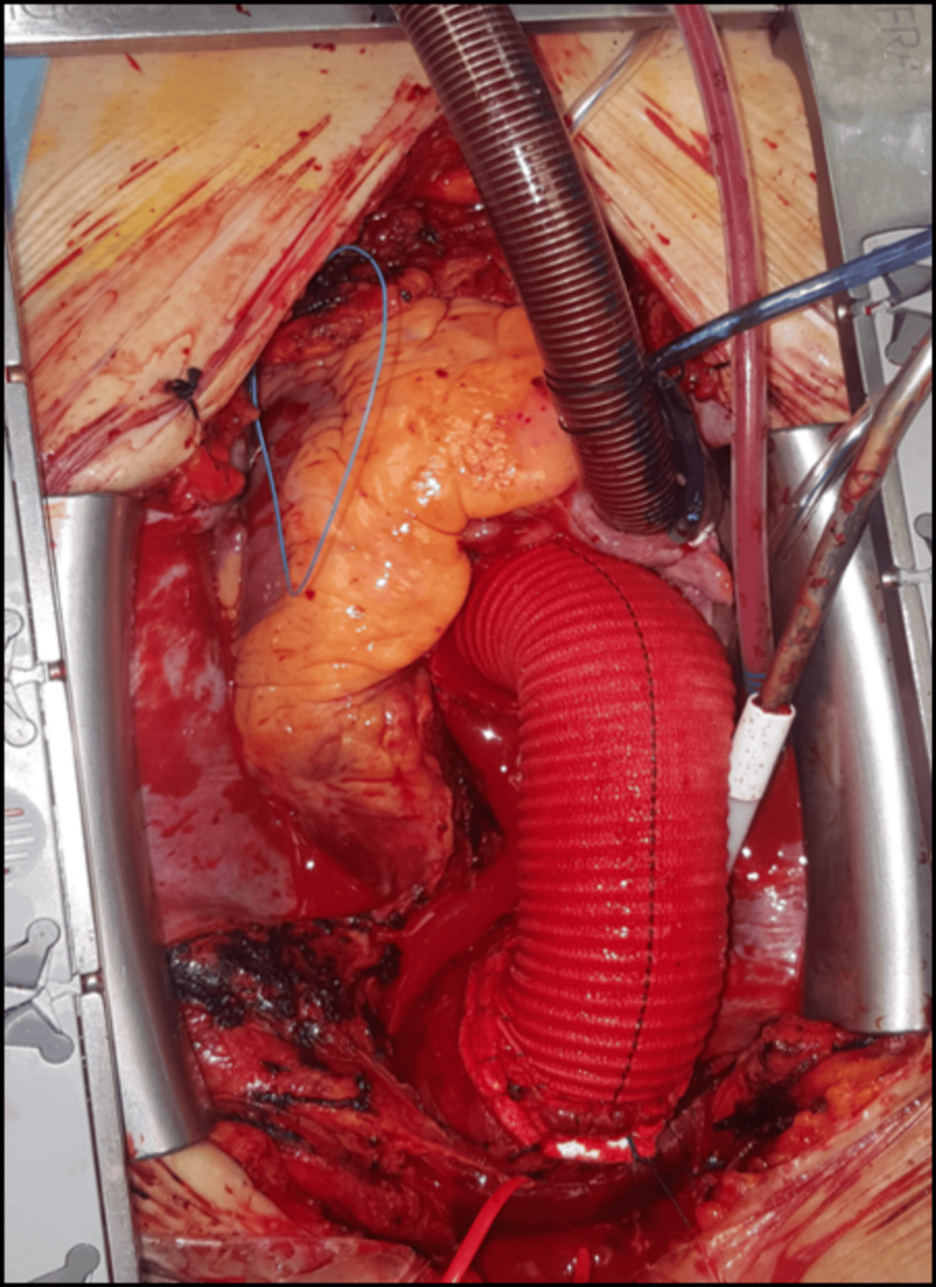
Final intraoperative result showing completed repair.

Intraoperative exploration revealed a dilated ascending aortic aneurysm with widespread hematoma involving the pulmonary artery, its branches, and the anterior surface of the heart. There was a spiral intimal tear, which extended from the sinotubular junction posteriorly, into the non-coronary sinus of Valsalva. Complete dissection of the ascending aorta was noted. The aortic valve demonstrated severe regurgitation. An adequate correction was questionable and therefore, due to the instability and high-risk profile of the patient, a valve replacement was deemed necessary. Following the establishment of cardiopulmonary bypass and total circulatory arrest under deep hypothermia at 24°C, the dissected ascending aorta was excised and replaced with a 28 mm Dacron synthetic graft. The regurgitant aortic valve was replaced with a 21 mm bioprosthetic valve and the graft anastomoses were supported with Teflon felt to increase effectiveness and decrease bleeding or rupture risks (Figure 3). The total ischemic time during the procedure was 132 minutes. He was successfully weaned off cardiopulmonary bypass without complications, and hemostasis was achieved before being transferred to the cardiac intensive care unit (ICU) in stable condition under moderate inotropic support.

Postoperative course

Postoperatively, the patient developed coagulopathy and required transfusions of blood products such as platelets, plasma, and clotting factors. Neurological recovery was slow, and brain CT was carried out on the fifth postoperative day, which showed no ischemic or hemorrhagic injury. Transesophageal echocardiography showed normal motion of the bioprosthetic valve and follow-up chest X-rays demonstrated a pericardial effusion, which was handled accordingly with a subxiphoid pericardial window in the ICU. He was transferred to the general ward on postoperative day 9 and discharged home on postoperative day 15 after stable course with no residual complaints.

Follow-up

**Figure 4:**
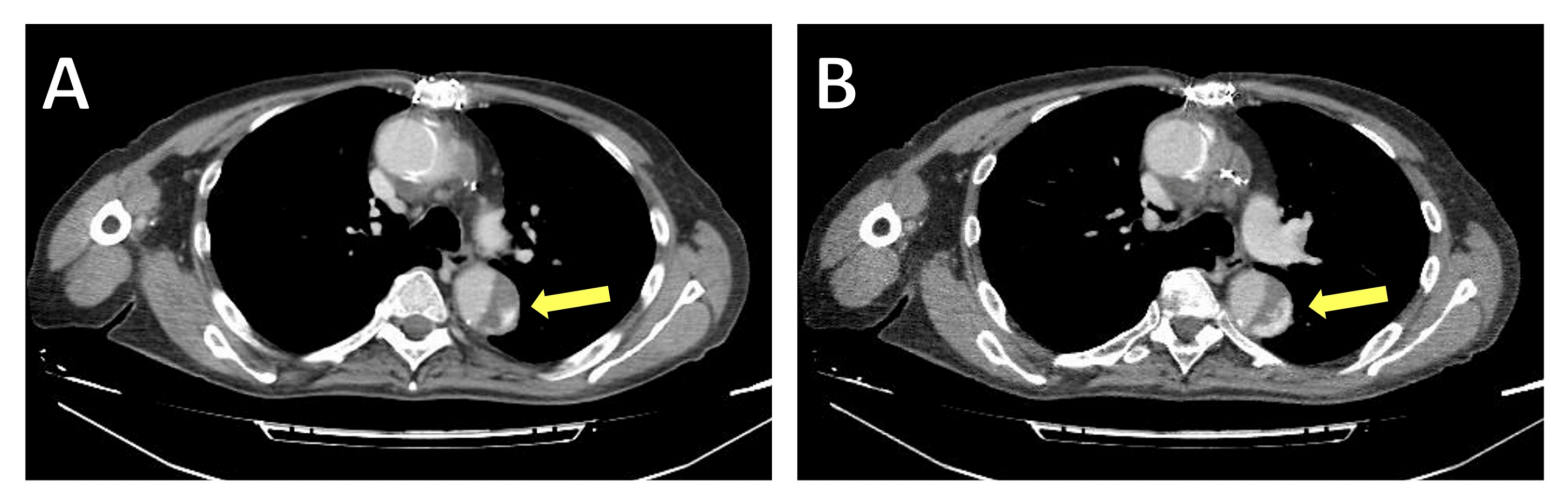
Follow-up chest CT findings: (A) First-month follow-up showing no pleural effusion and chronic dissection of the descending aorta, partially thrombosed (arrows), without significant dilatation. (B) Sixth-month follow-up confirming stability, with no pleural effusion and unchanged chronic dissection of the descending aorta (arrows).

At his one-month follow-up, chest CT demonstrated resolution of the pleural effusion and a stable, partially thrombosed chronic dissection of the descending aorta without further dilation. At six months, the patient remained asymptomatic, with no evidence of complications, indicating an excellent surgical and clinical outcome (Figure 4).

## Discussion

AAD is a medical and surgical emergency that requires quick decision-making in the presence of life-threatening complications. This case illustrates the vital challenges and dilemmas encountered while managing a patient with AAD complicated by out-of-hospital cardiac arrest. This successful outcome reflects the value of collaboration across specialties as well as a systematic approach to managing the unique challenges associated with these patients. The main dilemmas that arose during the decision-making and management of the patient are discussed below.

Dilemma 1: continuing or stopping resuscitation

Out-of-hospital cardiac arrest has been associated with high mortality rates and poor neurological outcomes, especially after prolonged resuscitation. Studies show that the chance of survival drops dramatically after 20 minutes of CPR and that neurological injury becomes increasingly likely [4]. In certain life-threatening, yet potentially reversible conditions, like AAD, aggressive resuscitation can lead to meaningful survival. Improved survival rates in these critically ill patients are significantly associated with early identification of reversible etiologies and persistence with CPR in the absence of pulseless electric activity [5].

In our patient’s case, the decision to continue resuscitation, despite the prolonged duration, was justified by the patient’s relatively young age and the potential reversibility of the underlying condition through surgical intervention. Despite evolving recognition of the association of anoxic cardiac arrest with AAD, some specific clinical aspects of such cases position the condition quite low in the differential diagnosis of cardiac arrest (for example relatively young age of the patient, absence of extensive asphyxic findings) and can discourage brave resuscitation attempt; the experience of a successful ROSC after prolonged CPR therefore underlines the importance of considering AAD in the differential diagnosis of cardiac arrest and implement resuscitation if potentially reversible.

Dilemma 2: coronary angiography before surgery

Differentiating between ACS and various other catastrophic conditions, such as AAD, is difficult in patients presenting with chest pain, cardiac arrest, and nonspecific electrocardiographic findings [6,7]. Coronary angiography is the standard for the diagnosis of coronary artery disease in a population with high clinical suspicion for left main coronary obstruction. Its role in suspected AAD, however, is debated. Some authors advocate that CT imaging needs to be prioritized by virtue of its swift ability to diagnose not only dissection but also reveal coronary involvement [8]. Others argue for non-elective angiography which can yield key data, particularly in those with equivocal results or hemodynamic instability, as well as upgrade patients’ clinical state by resolving serious complications [9].

Coronary angiography showed patent coronary arteries in our case; thus, ACS was excluded as the arrest cause. Most significantly, intimal flap and severe aortic regurgitation were identified, leading to the diagnosis of AAD. Although this part of the diagnostic process seemed to make it longer, it was necessary in directing further management. The positive outcome provides evidence in favor of selective angiography in patients with potential coronary involvement.

Dilemma 3: preoperative CT imaging

Computed tomography angiography (CTA) is considered the gold standard for AAD diagnosis and evaluation of the extent of dissection [7-9]. This is particularly important as studies have shown CTA to offer vital information regarding the anatomy of the dissection, involvement of branch vessels, and the presence of complications including pericardial effusion or malperfusion [10,11]. Nonetheless, among unstable patients, preoperative imaging must balance the potential advantages of elaborate anatomic information with the potential concern about delaying surgical intervention.

In this situation, total-body CT when combining thoracic with brain and abdominal imaging yielded critical data to determine the prognosis and plan the surgical treatment strategy. The CT findings confirmed the extent of the dissection, including the involvement of the abdominal aorta and branch vessels and associated pericardial and pleural effusions. Though imaging came with a short-added time delay, the insight gained in anatomy allowed highly tailored surgical/operative intervention as well as running intraoperative decision-making.

Dilemma 4: surgical approach and type of valve replacement

The surgical management of AAD involves replacing the dissected aorta with a synthetic graft and addressing associated complications, such as aortic valve dysfunction [12]. The choice of valve replacement-mechanical or bioprosthetic-depends on factors such as patient age, comorbidities, and long-term anticoagulation requirements. Guidelines generally recommend bioprosthetic valves for older patients, as they avoid the need for lifelong anticoagulation and its associated risks [13]. However, mechanical valves may be favored for their durability in younger patients.

In this case, a bioprosthetic valve was selected, given the patient’s age (68 years) and the uncertainty surrounding neurological recovery after prolonged arrest. This decision aligns with current guidelines and reflects a cautious approach to minimize potential complications. Using axillary cannulation and hypothermic circulatory arrest provided optimal conditions for surgical repair and reduced the likelihood of neurological complications [14], while reinforcement of graft anastomoses with Teflon felt ensured durability. These techniques are supported by the literature as standard practices in managing complex dissections.

Dilemma 5: postoperative management of neurological and hemodynamic recovery

Neurological injury is a major concern in patients undergoing prolonged CPR and complex cardiovascular surgery. Studies indicate that the incidence of hypoxic-ischemic brain injury increases with the duration of resuscitation [15], but early postoperative imaging can help stratify risk and guide prognosis. In this case, a brain CT performed on postoperative day 5 showed no ischemic or hemorrhagic lesions, providing reassurance regarding neurological recovery.

Postoperative coagulopathy is another common complication in extensive aortic surgery, often requiring aggressive management with blood products and clotting factors [16]. This patient required significant transfusion support, which is consistent with the literature. Additionally, the presence of a pericardial effusion, a recognized complication of AAD surgery, was successfully managed with subxiphoid drainage. These steps highlight the importance of vigilant postoperative monitoring and timely intervention to address complications.

Concluding remarks

This case highlights the importance of multidisciplinary management for complex AAD. Whether for resuscitation, surgical planning, or postoperative care, the risks and benefits were weighed with care according to evidence and clinical judgment. This case emphasizes the importance of persistence in resuscitation, timely imaging, advanced surgical techniques, and diligent postoperative care in ensuring good outcomes in a very dangerous pathology.

## Conclusions

AAD complicated by cardiac arrest is a life-threatening condition that is less common but should be considered as a possible diagnostic entity. This case highlights interdisciplinary collaboration, resuscitative tenacity, and timely application of diagnostic imaging to facilitate emergent surgical management. The successful outcome highlights the need for tailored surgical strategies, such as valve replacement and graft reinforcement, alongside vigilant postoperative care to address complications and ensure long-term stability. With coordinated care and advanced techniques, even high-risk cases of AAD can achieve favorable outcomes.
